# Increased risk of Enterococcal Bacteremia in critically ill patients with COVID-19 during pandemic surges

**DOI:** 10.1017/ash.2025.10056

**Published:** 2025-09-15

**Authors:** Sharanjeet K. Thind, Ghias N. Sheikh, Syeda Sahra, Dena R. Shibib, Awais Bajwa, Houssein A. Youness, Chris A. Gentry

**Affiliations:** 1 Section of Infectious Diseases, Medical Service, Oklahoma City VA Healthcare System, Oklahoma City, OK, USA; 2 Department of Medicine, University of Oklahoma Health Sciences Center, Oklahoma City, OK, USA; 3 Section of Pulmonary, Critical Care and Sleep Medicine, Oklahoma City VA Healthcare System, Oklahoma City, OK, USA; 4 Department of Pathology, Oklahoma City VA Health Care System, Oklahoma City, OK, USA; 5 Department of Pathology, University of Oklahoma Health Sciences Center, Oklahoma City, Oklahoma, USA; 6 Pharmacy Service, Oklahoma City VA Health Care System, Oklahoma City, OK, USA

## Abstract

**Objective:**

Identify incidence of enterococcal bacteremia and associated risk factors in ICU patients with SARS-CoV-2 infection.

**Design:**

Retrospective cohort study.

**Setting:**

Veterans Affairs Medical Center, Oklahoma City, Oklahoma.

**Patients:**

Adult ICU patients with SARS-CoV-2 and positive blood cultures for *Enterococcus* species between March 1, 2020 and February 28, 2022.

**Methods:**

Durations of hospitalization, ICU stay, ventilation, site and duration of central line, receipt of steroids, tocilizumab, and antimicrobials were gathered by chart review. Patient location during a surge of bacteremia was noted and strains were identified by multilocus sequencing typing (MLST).

**Results:**

There were 70 episodes of enterococcal bacteremia in ICU patients with COVID-19 during a 2-year period. Patients had a median age of 72 years and 97% were male. Onset of bacteremia was 12 and 14 days after mechanical ventilation and central line placement, respectively. The median number of days to bacteremia diagnosis was 13 from admission to the ICU and 90-day mortality was 66.7% among patients admitted October to December of 2020. A large proportion of ICU patients developed enterococcal bacteremia during a COVID-19 surge (*P* < .00001) and an increased incidence of enterococcal bacteremia was seen September 2020—February 2021 (*P* < .0001) in hospitalized patients. A total of 5 unique Enterococcal strains among 13 bacteremia episodes were identified in patients with ICU beds in close proximity.

**Conclusions:**

A high incidence of enterococcal bacteremia was observed in critically ill patients with SARS-CoV-2, especially during surges. Contributing factors may include environmental contamination, patient colonization, nonadherence to infection control practices, resource limitations, ICU design and use of mechanical ventilation, central lines and immunosuppressants. MLST can be used to identify clusters to address these contributing factors.

## Introduction

An unprecedented burden of morbidity and mortality has been observed in patients admitted to healthcare facilities with moderate to severe COVID-19 infection globally since March 2020. Important contributing factors are the presence of concurrent or superimposed bacterial infection. Prolonged hospitalization, invasive mechanical ventilation and presence of central venous catheters increase the risk of hospital-acquired infections including bacterial pneumonia and bloodstream infections (BSIs) with a significant proportion that are due to antimicrobial resistant pathogens.^1,2^ At the beginning of the pandemic, rates of bacterial coinfection were described to be less than 10%, similar to previous studies on severe acute respiratory syndrome (SARS) and Middle East respiratory syndrome (MERS).^3^ One meta-analysis showed an even lower rate of coinfection at 7% in hospitalized patients and up to 14% in critically ill patients in the intensive care unit (ICU) arguing against widespread use of antibiotics.^4^ Despite this, 80% of patients received antimicrobials; mostly broad-spectrum agents with anti-MRSA activity.^5^

A study by Garcia–Vidal et al described 51 hospital-acquired bacterial infections in patients with COVID-19 that were mainly caused by *Pseudomonas aeruginosa* and *E. coli, Klebsiella pneumoniae* and *Staphylococcus aureus* with a mean time from hospitalization to superinfection diagnosis of 10.6 days with an overall mortality of 9.8%.^6^ In early 2021, a meta-analysis of 154 studies showed a coinfection rate of 8.6%. The most common infectious complication were pneumonia and BSIs. Risk factors for bacterial infections were identified as steroid use, indwelling catheters, and prolonged mechanical ventilation.^7^ Subsequent meta-analyses have reports similar bacterial co-infection rates of 11%.^8,9^

In patients who were critically ill and had bacterial infections, BSIs (34% of cases) were the second most common diagnosed infection after pneumonia (50% of cases).^10^ In some studies, as many as 40% of patients infected with COVID-19 in the ICU acquired bloodstream infections with a cumulative risk increase with the length of ICU stay, use of steroids and anti-inflammatory agents including tocilizumab, and indwelling catheters.^11,12^ In the study by Grasselli et al., most bloodstream infections were due to gram-positive bacteria (54%), mainly enterococci, followed by *Staphylococcus aureus* and coagulase-negative staphylococci (CoNS).^10^

In a study from a quaternary care hospital in San Francisco, California, COVID-19 patients were noted to have a higher incidence of BSI secondary to enterococcal species and whole genome sequencing of enterococcus isolates did not explain the transmission. Eight out of 314 patients (2.55%) hospitalized with COVID-19 infection had enterococcal BSIs, with an even higher incidence of 6.4% in those admitted to the ICU (8/126). This was much higher than the incidence in the hospitalized control group (0.3%) and ICU control group (0.7%).^13^

In a study by Cataldo et al., bloodstream infections were noted in half of patient’s admitted to the MICU with COVID-19 with the mean time from MICU admission to occurrence of BSIs being 13 ± 7 days and the most common isolated pathogens were *Enterococcu*s species and *Pseudomonas aeruginosa*. Of the *Enterococcus* species, 20% were noted to be vancomycin-resistant enterococci (VRE). A quarter of this cohort acquired a subsequent bloodstream infection during the ICU stay onwards.^14^ Similarly, a study of 89 COVID-19 patient from an ICU in Milan, Italy showed an incidence of 67.4% of bacteremia with median time from ICU admission to first bloodstream infection of 10 days. Approximately 80% of the bacteremias were noted to be secondary to gram-positive bacteria with *Enterococcus* species accounting for 55.8% of the cases.^15^ Kampmeier et al reported a nosocomial vancomycin-resistant enterococci outbreak in COVID-19 ICU where whole genome sequence based typing revealing 2 genotypically distinct VRE clusters.^16^

Given the large number of enterococcal infections that was being observed in association with SARS-CoV-2 infection and prevalence of resistant strains, we aimed to identify host-related, and environmental risk factors associated with enterococcal bacteremia in critically ill patients hospitalized with SARS-CoV-2 infection. In addition, we traced the strains of enterococcal species in a group of patients in the ICU to determine factors such as infection control (IC) practices and ICU design that could have contributed to the increase in enterococcal bacteremia that was observed during surges of SARS-CoV-2 infection at our hospital.

## Methods

We performed a retrospective cohort study of patients with SARS-COV-2 infection in the ICU who were also identified as having at least one positive blood culture result for enterococcal species on or after a SARS-CoV-2 positive test, between September 1, 2020 to February 28, 2022. Data was gathered by review of patient records using the Computerized Patient Record System (CPRS). We collected pertinent data including duration of ICU stay, body mass index (BMI), duration and site of central line placement, duration of ventilation, receipt of steroids, remdesivir, tocilizumab and antimicrobials. We also identified the ICU unit and room location during the diagnosis of bacteremia spanning a three-month period from October 1, 2020 to December 31, 2020. In these patients multilocus sequence typing (MLST) was performed to identify enterococcal species strain, detailed methods are provided in the Appendix.

Additionally, we reviewed hospital epidemiology data for an additional 6 months prior to the study period to demonstrate the number of patients that were hospitalized with SARS-CoV-2 infection during a two-year period from March 1, 2020 to February 28, 2022 as well as the number of patients who were diagnosed with enterococcal bacteremia during that time. Furthermore, we compiled data on the number of blood culture sets that were collected during the same two-year period and the number of blood culture sets that were positive for enterococci. Approval of this retrospective study was obtained from the University of Oklahoma Health Science Center Institutional Review Board and Oklahoma City Veterans Affairs (VA) Healthcare System Research and Development Committee with waiver of patient consent (protocol 13 454).

### Statistical analysis

For all tests and analyses, an a-priori level of significance was set at *p* ≤ .05. Categorical variables were assessed using Chi-squared test. The Cochran-Armitage test was used to assess trends.

## Results

There was a total of 70 episodes of enterococcal bacteremia diagnosed in patients admitted to the ICU during a 2-year period from March 1, 2020 to February 28, 2022. There was a total of 35 episodes of enterococcal bacteremia among 33 patients admitted to the ICU from September 1, 2020 to Feb 28, 2022 that had sufficient data for chart review. Thirty-four of these episodes were associated with a positive test for SARS-CoV-2 within 30 days of the date of positive blood culture. One patient did not have a positive SARS-CoV-2 test result.

The median age of the patients was 72 years and 97% (32/33) of patients were male with a median BMI of 30.7. The diagnosis of bacteremia was made a median of 11 days after the initial positive SARS-CoV-2 test and after a median of 13 days in the ICU. Most episodes—65% (23/35) were associated with mechanical ventilation and patients that were intubated were done so for 12 days before the bacteremia was detected. Most patients -60% (21/33) were proned for at least 1 day. Central lines were in place for a median of 13.5 days and the most common site was right internal jugular vein (15/35 episodes). The remaining episodes of bacteremia were associated with multiple central lines (3), left internal jugular line (5), hemodialysis catheter (1), peripherally inserted central catheter (2). Patient characteristics and risk factors for enterococcal bacteremia are presented in Table 1.

**Table 1.** Patient characteristics and risk factors for Enterococcal bacteremia

Bacteremia case (*n* = 35)	Age (years)	Sex (M/F)	BMI (Kg/m2)	APACHE score on ICU admission	Bacteremia onset after positive SARS-CoV-2 test (days)	ICU LOS prior to bacteremia onset (days)	Central line days	CVC site	Ventilation days	Prone (Y/N)	Antimicrobial days prior to bacteremia onset	Remdesivir (Y/N)	Steroids prior to bacteremia onset (Y/N)
**1**	87	M	26.1	20	6	3	16	PICC	–	N	7	N	N
**2**	86	M	31.5	19	9	9	8	R SCV	6	Y	9	Y	Y
**3**	85	M	30.0	17	17	18	18	R IJ	18	N	16	Y	Y
**4**	81	F	36.4	26	13	15	14	R IJ	14	Y	11	Y	Y
**5**	70	M	24.9	17	3	1	–	PIV	–	N	2	Y	Y
**6**	73	M	41.2	18	12	12	11	R IJ	11	Y	5	Y	Y
**7**	75	M	26.4	20	14	13	11	L IJ	11	Y	9	Y	Y
**8**	72	M	27.9	20	10	11	11	R IJ	11	Y	4	Y	Y
**9**	73	M	45.4	23	2	19	18	Multiple	18	Y	9	Y	Y
**10**	74	M	26.6	23	18	14	17	L IJ	5	Y	8	Y	Y
**11**	81	M	26.4	14	10	0	–	PIV	–	N	1	N	Y
**12**	75	M	29.5	12	8	8	7	R IJ	7	Y	9	Y	Y
**13**	71	M	35.2	8	16	15	8	R IJ	8	Y	8	Y	Y
**14**	75	M	31.4	7	0	13	13	R IJ	13	Y	8	Y	Y
**15**	82	M	34.7	11	6	10	2	R IJ	–	N	5	Y	Y
**16**	67	M	58.4	10	0	0	–	PIV	–	N	–	Y	N
**17**	83	M	26.0	11	4	NA	–	PIV	–	N	1	N	Y
**18**	59	M	35.1	14	27	39	27	Multiple	26	Y	21	N	Y
**19**	58	M	33.5	25	19	2	–	HD	–	N	16	N	Y
**20**	60	M	33.3	9	30	31	18	Multiple	18	Y	14	Y	Y
**21**	76	M	33.5	24	13	14	14	R IJ	14	Y	6	Y	Y
**22**	69	M	30.7	28	0	0	59	PICC	–	N	11	N	Y
**23**	66	M	46.0	17	1	8	9	R IJ	8	Y	13	Y	Y
**24**	62	M	30.4	20	12	21	23	R IJ	22	Y	11	Y	Y
**25**	69	M	29.0	14	5	NA	–	PIV	–	N	4	N	N
**26**	68	M	29.6	22	17	17	18	R IJ	17	Y	7	N	Y
**27**	74	M	35.1	16	11	12	12	L IJ	12	Y	7	N	Y
**28**	55	M	16.0	8	0	NA	–	PIV	–	N	–	N	N
**29**	51	M	36.1	12	11	12	8	R IJ	8	Y	5	Y	Y
**30**	63	M	27.8	12	27	21	16	R IJ	16	Y	20	Y	Y
**31**	67	M	19.2	^*^	^**^	NA	–	PIV	–	N	–	N	N
**32**	76	M	33.8	15	43	33	31	R IJ	26	Y	17	Y	Y
**33**	76	M	31.7	15	6	0	–	PIV	–	N	4	Y	Y
**34**	72	M	26.1	18	25	18	5	L IJ	9	Y	18	Y	Y
**35**	72	M	26.1	18	27	20	7	L IJ	11	Y	20	Y	Y

M, male; F, female; BMI, body mass index; kg, kilogram; m^2^, square meter; APACHE, Acute Physiology and Chronic Health Evaluation; SARS-CoV-2, sudden acute respiratory syndrome coronavirus-2; ICU, intensive care unit; LOS, length of stay; PICC, peripherally inserted central catheter; R, right; L, left; SCV, subclavian vein; IJ, internal jugular vein; PIV, peripheral intravenous; HD, hemodialysis; Y, yes; N, no.

* Not admitted to ICU.

** Negative SARS-CoV-2 test.

Most patients (30/33) received broad-spectrum antibacterials and antifungals prior to the bacteremia episode, 3 patients did not. Most patients received 2 or more agents; 28 patients received a cephalosporin, 18 patients received vancomycin, 16 received azithromycin, 15 received piperacillin/tazobactam and 11 received a fluoroquinolone as a part of their antimicrobial regimen. Other antimicrobials received included daptomycin, ampicillin/sulbactam, linezolid, carbapenems, doxycycline, fluconazole, micafungin, and metronidazole.

Out of the 33 patients, 22 received remdesivir, 29 received steroids, most commonly dexamethasone, and for a median duration of 10 days during their hospitalization. The median total hospital length of stay among 33 patients was 31 days. The 90-day mortality was 66.7% (22/33) with no difference in mortality observed in patients who had a BSI from *Enterococcus faecium* versus *Enterococcus faecalis* (Table 2).

**Table 2.** Enterococcal species, total length of stay and 90-day all-cause mortality

Bacteremia case (*n* = 35)	Blood culture result	VRE or S to vancomycin	Total hospital LOS (days)	90-day all-cause mortality
**1**	*E. faecalis*	S	32	Y
**2**	*E. faecalis*	S	31	Y
**3**	*E. faecalis*	S	21	Y
**4**	*E. faecalis*	S	16	Y
**5**	*E. faecalis*	S	74	N
**6**	*E. faecium*	VRE	18	Y
**7**	*E. faecalis*	S	23	Y
**8**	*E. faecalis*	S	34	N
**9**	*E. faecium*	VRE	118	N
**10**	*E. faecium*	VRE	56	Y
**11**	*E. faecium*	VRE	16	Y
**12**	*E. faecium*	VRE	56	N
**13**	*E. faecium*	VRE	16	Y
14	*E. faecium/E.faecalis*	VRE/S	28	Y
**15**	*E. faecalis*	S	48	Y
**16**	*E. faecalis*	S	47	Y
**17**	*E. faecalis*	S	13	Y
**18**	*E. faecium*	VRE	39	Y
**19**	*E. faecalis*	S	100	N
**20**	*E. faecium*	VRE	43	Y
**21**	*E. faecium*	VRE	69	N
**22**	*E. faecium*	VRE	4	Y
**23**	*E. faecium*	VRE	28	Y
**24**	*E. faecalis*	S	22	Y
**25**	*E. faecium*	VRE	15	N
**26**	*E. faecalis*	S	72	N
**27**	*E. faecalis*	S	27	Y
**28**	*E. faecalis*	S	7	N
**29**	*E. faecalis*	S	15	Y
**30**	*E. faecium*	VRE	46	Y
**31**	*E. faecalis*	S	10	N
**32**	*E. faecium*	VRE	92	N
**33**	*E. faecium*	VRE	92	N
**34**	*E. faecalis*	S	31	Y
**35**	*E. faecium*	VRE	31	Y

E, enterococcus; VRE, vancomycin-resistant enterococci; S, susceptible to vancomycin; LOS, length of stay; Y, yes; N, no.

Most bloodstream infections were monomicrobial, 7 (20%) were polymicrobial. Out of 35 episodes that included an *Enteroccocus* species, 19 were due to *E. faecalis*, 1 of which was a VRE, 15 were due to *E. faecium*, all of which were VRE and 1 case of bacteremia included both *E. faecalis* (VRE) and *E. faecium* (susceptible to vancomycin). Other pathogens isolated in polymicrobial bacteremia included *S. aureus*, CoNS (*S. epidermidis*, *S. hominus*, *S. haemolyticus* and others), *Enterococcus raffinosus*, and *Granulicatella adiacens*.

Twenty-two episodes of enterococcal bacteremia occurred among 20 patients in the ICU during a 3-month period from October 1, 2020 to December 31, 2020. Out of these, 13 episodes were due to Enterococcal strains that were isolated in at least one other case of bacteremia from a different patient. Of these episodes, 6 were due to *E. faecalis* and 7 due to *E. faecium*.

Of the 10 *E. faecalis* isolates, there were 7 different strains. Strains identified as MLST 1 112, MLST 64, MLST 6 were each isolated in two bacteremia episodes. Sequence type 1 111 and 1 112 were not previously reported in PubMLST and were added to the database. Sequence type 16/865 could not be identified because 1 of the 6 genes could not be amplified. Of the 12 *E. faecium* isolates, there were 7 different strains. The strain identified as MLST 1897 was associated with 4 different episodes of bacteremia and strain identified as MLST 1901 was isolated in 3 bacteremia episodes (Table 3).

**Table 3.** *E. faecalis* and *E. faecium* strain identification based on MLST

*E. Faecalis* Strain Typing by MLST								
PHRL ID	Allele Type							MLST ID
	gdh	gyd	pstS	gki	aroE	xpt	yqiL	
66	5	1	1	3	106	1	6	1 111
67	9	7	3	2	6	1	5	1 112
83	9	7	3	2	6	1	5	1 112
68	5	1	1	3	7	1	6	179
72	12	7	3	32	32	26	31	778
73	10	1	11	6	5	1	4	64
85	10	1	11	6	5	1	4	64
74	5	1	1	No Amp	7	7	6	16/185
84	12	7	3	7	6	1	5	6
82	12	7	3	7	6	1	5	6
***E. Faecium* Strain Typing by MLST**								
PHRL ID	Allele Type							MLST ID
	atpA	ddl	gdj	purK	gyd	pstS	adk	
63	74	1	1	1	1	1	1	1895
64	74	1	1	1	2	51	1	1896
65	74	1	1	1	12	1	1	1897
70	74	1	1	1	12	1	1	1897
75	74	1	1	1	12	1	1	1897
77	74	1	1	1	12	1	1	1897
69	74	1	1	44	1	20	1	1898
76	52	1	1	1	12	1	1	1901
78	52	1	1	1	12	1	1	1901
79	52	1	1	1	12	1	1	1901
80	47	1	1	1	12	1	1	1905
81	52	1	1	1	44	3	1	1906

PHRL, Public Heath Reference Library; ID, identification; MLST, Multilocus sequence type; No Amp, No amplification.

**Figure 1.**
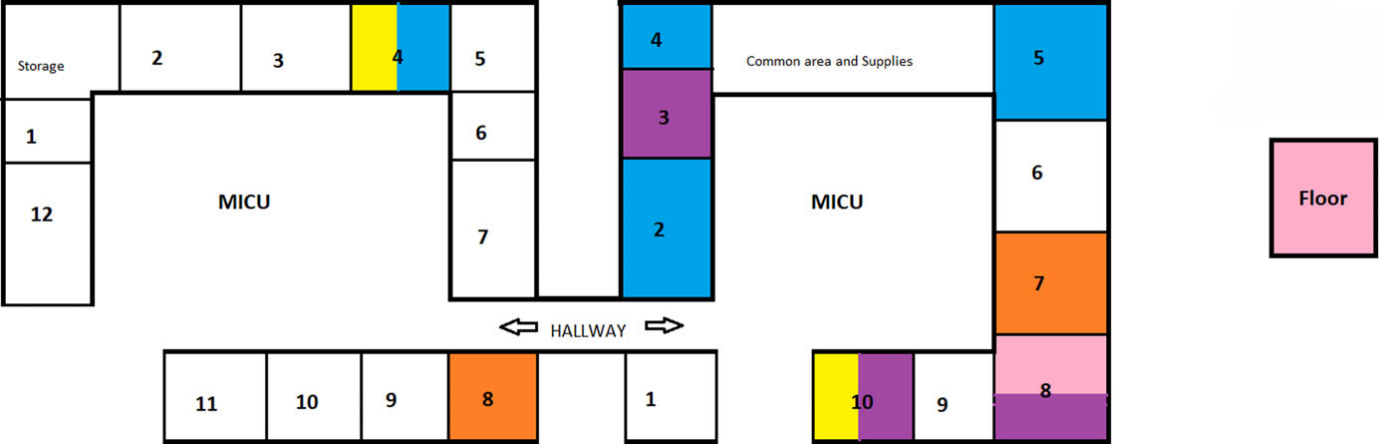
Geographical representation of patients located in the ICU that developed Enterococcal BSIs from specific enterococcal strains. Each color represents a unique strain.

There was a total of 13 episodes of bacteremia that were caused by a total of 5 unique strains of *Enterococcus sp.* The patients that shared identical strains were located in close proximity to each other in the ICU and their onset of bacteremia was within 30 days of each other except for one pair of patients that shared an identical strain but were not in close geographic proximity and had an onset of bacteremia that was 48 days apart (Figure 1).

**Figure 2.**
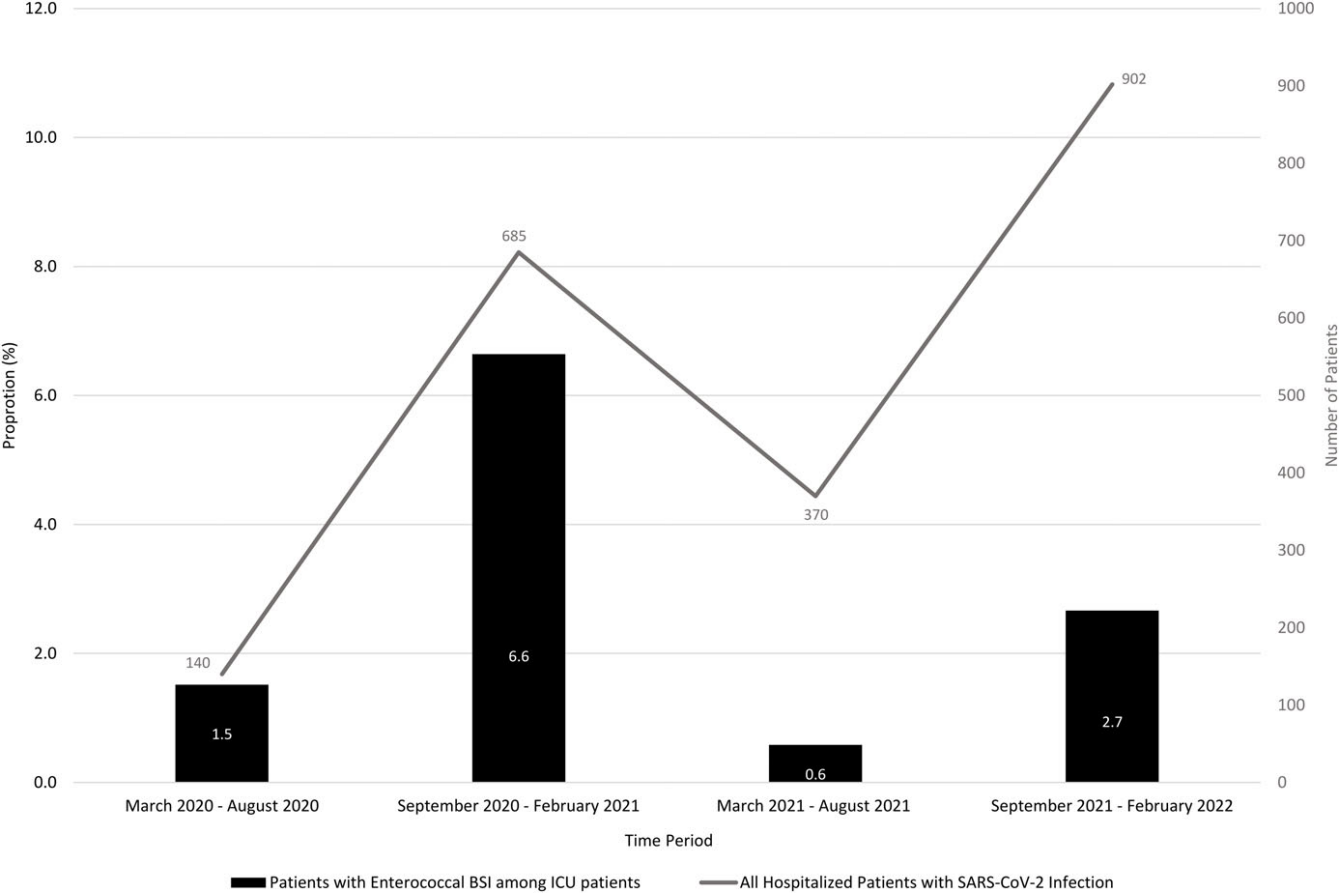
Illustrates the number of patients hospitalized with SARS-CoV-2 infection (line) and the proportion (bar) of patients with both SARS-CoV-2 and Enterococcal BSIs among the ICU population over four, six-month time intervals.

Episodes of enterococcal bacteremia in SARS-CoV-2 positive patients were high during two specific six-month period during which there was a surge in COVID-19 related hospitalizations. The timespan from September 2020 to February 2021 had significantly more associated enterococcal episodes of bacteremia than the other time periods (Figure 2). There was significantly large proportion of patients who developed enterococcal bacteremia in the ICU during the first surge in COVID-19 hospitalizations compared to other time periods (*P* value is <.00001, Chi-squared test).

**Figure 3.**
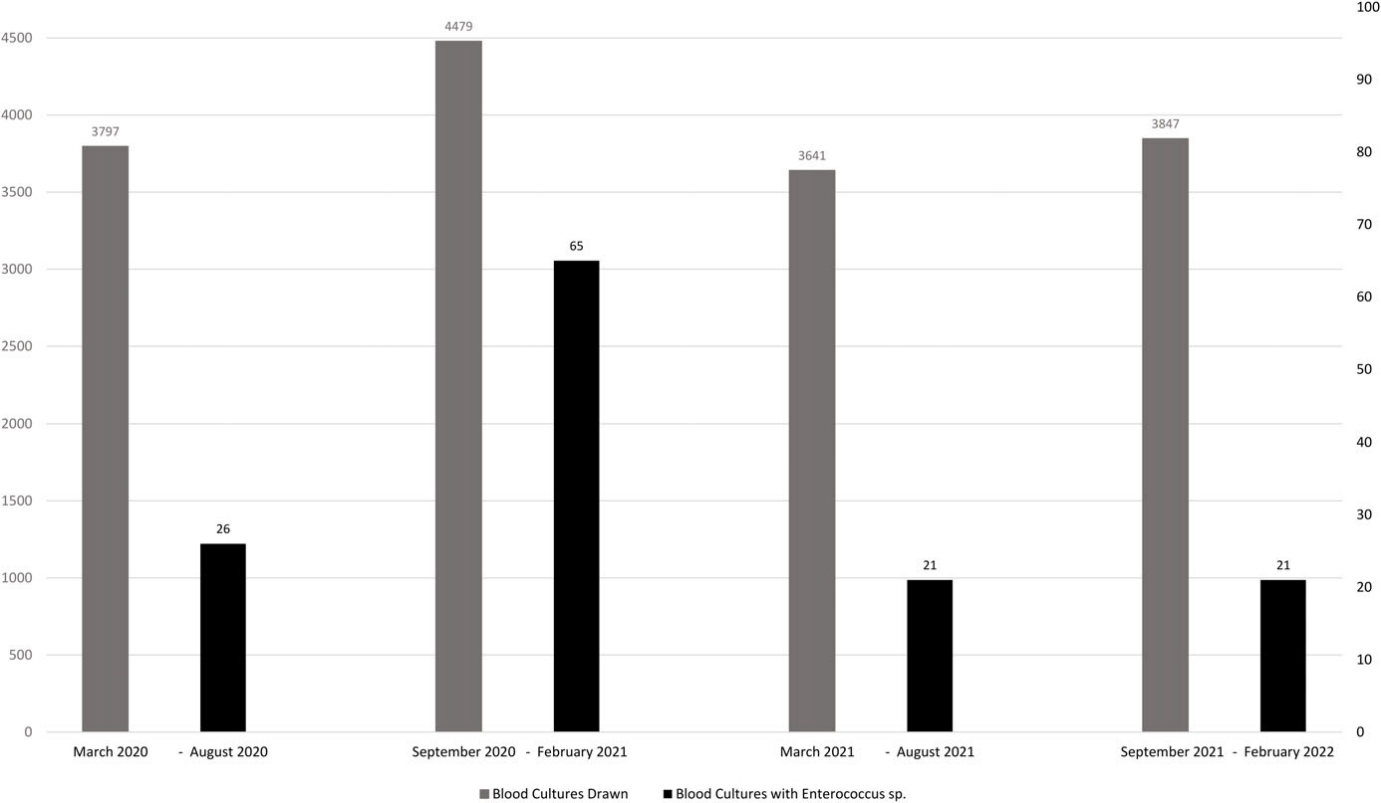
Shows an increase in the number of blood cultures drawn and increase in those positive for *Enterococcos* species among hospitalized patients during COVID-19 surges over two years.

A total of 15 764 blood culture sets were collected from March 1, 2020, to February, 2022, out of which 133 sets were positive for *Enterococcus* species. There was an increased incidence of positive blood culture sets with *Enterococcus species* among all hospitalized patients from September 2020 to February 2021 compared to the other three six-month time periods (likelihood ratio 25.629, *p* = <.0001, Cochran-Armitage trend test *Z* = 3.88, *p* = <.0001. There was a significant increase in hospitalizations due to SARS-COV-2 during the same time period (Figure 3).

## Discussion

There has been significant accrual of evidence confirming an increase in BSIs including central line-associated bloodstream infections (CLABSIs) in the United States especially early in the COVID-19 pandemic. Multiple factors have been identified in association with BSIs in healthcare settings such as patients with severe SARS-CoV-2 infection. Patient-related factors include prone positioning, increased BMI and comorbidities, presence and duration of indwelling catheters, depressed immune system secondary to immunomodulating and anti-inflammatory drugs including tocilizumab and dexamethasone.^11,13,15^ In our study, the median LOS in the ICU to bacteremia diagnosis was 13 days, and 90-day mortality was 66.7% among patients admitted October to December of 2020. The days to onset of bacteremia is similar to a study that showed that healthcare-associated infections (HAI) with BSI being the second most common HAI, occurred after a median of 12 days in the ICU.^10^ The mortality rate at our hospital was higher than a case series of critically ill patients with COVID-19 infections in the ICU. Their reported mortality was 42% and 57% at 30 days due to enterococcal BSI and VRE BSI respectively.^17^ A higher mortality may reflect our older population and more comorbid conditions (median age 72 vs 63 in this case series).^17^ In addition, studies have shown that alteration in IC practices to accommodate more patients in the hospital, decreased face to face contact, decreased compliance with central line care bundles, especially with patients that were proned, likely played a role.^18,19^

A high proportion of BSIs and infections due to multidrug-resistant organisms (MDRO)s including MRSA and VRE have also been associated with COVID-19 surges. In addition, clusters and outbreaks of HAIs have been identified in association with surges and are associated with increase mortality and some contributing factors have been identified.^1,20^ In one study by Afzal and colleagues there was increase in frequency of enterococcal positive blood cultures from 7.5 % in the two months preceding the pandemic to 22.5% in early pandemic—March 2020 to May 2020.^21^

A high proportion of patients with SARS-CoV-2 infection that developed enterococcal bacteremia at our hospital had several of the mentioned risk factors for BSIs and an increase in incidence of enterococcal bacteremia was clearly associated with a surge in COVID-19 cases during the time periods of September 2020 to February 2021 and September 2021 to February 2022. The significant increase in enterococcal bacteremia episodes was more prominent during the earlier surge, reflecting issues from the early part of the pandemic that may have been subsequently resolved.

Early in the COVID-19 pandemic, there was limited guidance on how to manage the novel, unpredictable respiratory virus. This, along with rapidly changing protocols and recommendations, shortage and re-use of personal protective equipment (PPE) especially during case surges led to a discrepancy in how IC precautions were enforced. Concern for healthcare worker safety and for transmission within the hospital led to cohorting of patients that were infected with SARS-CoV-2 to specific areas and assigned to specific healthcare workers (HCWs), namely increasing the burden to nursing staff that took on more patients than usual. HCW shortages due to illness and logistic complications during the pandemic further worsened this burden and intern made activities such as appropriately donning and doffing PPE prior to patient contact more difficult. Deployment of HCWs from other regions of the hospital to take care of ICU patients to mitigate shortages added more challenges and impacted the adherence to infection control measures.^19^

In addition to patient related factors, various environmental factors including limited room and surface cleaning due to rapid patient turnover, poor surveillance mechanisms and ICU design may effect rates of HAIs.^22^ The open-ICU design at our hospital during the surge in September 2020 to February 2021 could have contributed to the transmission of identical enterococcal strains to patients in the area due to environmental and/or shared device contamination such as ultrasounds used to place central lines and directly by healthcare workers. At our hospital, the increase in enterococcal bacteremia during the second surge period (September 2021 to February 2022) is less dramatic likely due to improvement in PPE accrual, availability of guidance for infection control protocols, vaccine availability, anti-viral treatments as well as education that we provided to ICU staff. In addition during this second surge, the omicron strain was the most predominant and studies have shown decreased need for intubation, dexamethasone, remdesivir, and shorter ICU length of stay leading to a reduced risk of BSIs compared to other strains. In addition, there was a lower mortality observed in patients hospitalized during the early omicron surge in early 2022.^23^

After an increase in enterococcal bacteremia cases were observed, our hospital’s infection control team, and nursing supervisors and managers discussed actions that could be implemented to prevent BSIs in patients with SARS-CoV-2 infection. A team of practitioners rounded daily to address central line indication, integrity of dressing and frequency of changes, indication, duration and upkeep of indwelling urinary catheters, monitoring of PPE donning and doffing methods, and hand hygiene. Use of hospital appropriate disinfectant and UV light on all terminal room cleanings and use of a checklist for high-touch surfaces including beds, bathrooms and floors was encouraged and specific training was provided. Shared spaces such as break rooms, and nursing station were also thoroughly cleaned with cleaning agents active against SARS-CoV-2 . These changes also likely contributed to a less pronounced surge in enterococcal bacteremia during the latter surge period. Implementation of similar interventions such as hand hygiene, cleaning skin with chlorhexidine and using full-barrier precautions during the insertion of central venous catheters, avoiding femoral lines and promptly removing unnecessary catheters among 103 ICUs in Michigan showed significant reduction (up to 66%) in bloodstream infections in the ICU.^24^

We had several study limitations including those associated with a retrospective study. We included all patients who had at least one blood culture set positive for enterococcus species and understand that this could represent contamination in some cases. In addition, we did not assess appropriateness or timeliness of antibiotics to treat the enterococcal bacteremia.

Overall, our study showed a high incidence of enterococcal bacteremia in critically ill patients infected with SARS-CoV-2 and a high mortality. This was more pronounced during COVID-19 surges. Several environmental factors such as surface colonization, nonadherence to infection control practices, open- ICU design, host factors such as mechanical ventilation, central lines and immunosuppressant use play a role. Potential resource limitations early in the pandemic and during surges likely contribute. MLST is an accepted tool for comparing strains among a group of isolates and yields good discriminative power. As such, strain identification by MLST can help recognize clusters in the hospital, and help identify and address contributing risk factors such as spread of organisms in a shared environment, by colonized devices or directly and unintentionally by HCWs. In addition, in this critically ill COVID-19 patient population, infections with enterococcal species including VRE was high and risks and benefits of starting empiric therapy should be considered. Individual patient rooms with dedicated donning/doffing areas, planned education and observation of healthcare workers, and ensuring quality of terminal cleaning may lead to decreased unintentional infection transmission.

## Supporting information

10.1017/ash.2025.10056.sm001 Thind et al. supplementary material Thind et al. supplementary material

## Supplementary material

The supplementary material for this article can be found at https://doi.org/10.1017/ash.2025.10056.

## Footnote

Some of the data contained in this manuscript was presented at the SHEA Spring Conference in Houston, TX in April, 2024.
