# Supplementary material for: Increased risk of Enterococcal Bacteremia in critically ill patients with COVID-19 during pandemic surges
Source: Antimicrob Steward Healthc Epidemiol. 2025 Sep 15;5(1):e215. doi: 10.1017/ash.2025.10056 (PMC12451795; doi:10.1017/ash.2025.10056)
Supplement: Thind et al. supplementary material [file S2732494X25100569sup001.docx]

**Appendix:**

DNA was isolated from each isolate and multi locus sequence typing was performed by amplifying 7 regions of E faecalis genome and 7 regions of E faecium genome by PCR. The resulting amplicons were sequenced and allele type for each gene region and overall sequence type (ST) was determined using the MLST database <http://pubmlst.org>). The results of genetic typing by multilocus sequenced typing of enterococcal bacterial strains were processed by public health reference laboratory, Palo Alto and were provided to Oklahoma City via microbiology lab.
